# Mutations at the M2 and M3 Transmembrane Helices of the GABA_A_Rs α_1_ and β_2_ Subunits Affect Primarily Late Gating Transitions Including Opening/Closing and Desensitization

**DOI:** 10.1021/acschemneuro.1c00151

**Published:** 2021-06-08

**Authors:** Katarzyna Terejko, Michał A. Michałowski, Ilona Iżykowska, Anna Dominik, Aleksandra Brzóstowicz, Jerzy W. Mozrzymas

**Affiliations:** † Department of Biophysics and Neuroscience, Wrocław Medical University, ul. Chałubińskiego 3A, 50-368 Wrocław, Poland; ‡ Department of Molecular Physiology and Neurobiology, University of Wrocław, ul. Sienkiewicza 21, 50-335 Wrocław, Poland

**Keywords:** Desensitization, GABA_A_ receptor, gating, transmembrane domain, opening/closing transitions, patch clamp

## Abstract

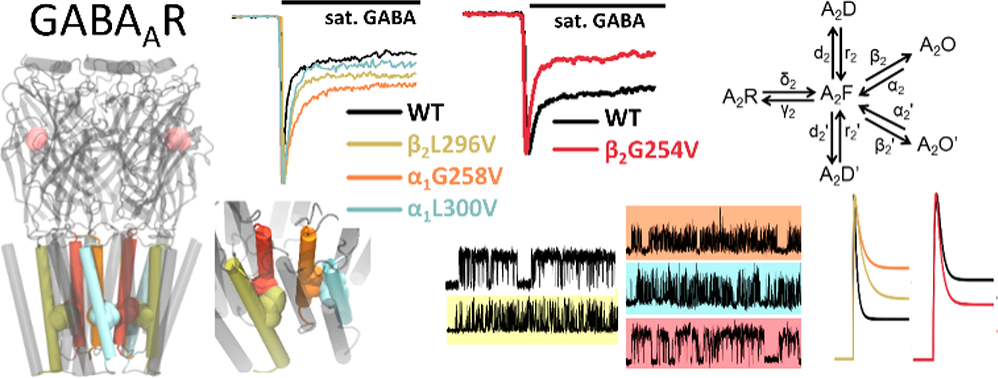

GABA type A receptors (GABA_A_Rs) belong to the pentameric ligand-gated ion channel (pLGIC) family and play a crucial role in mediating inhibition in the adult mammalian brain. Recently, a major progress in determining the static structure of GABA_A_Rs was achieved, although precise molecular scenarios underlying conformational transitions remain unclear. The ligand binding sites (LBSs) are located at the extracellular domain (ECD), very distant from the receptor gate at the channel pore. GABA_A_R gating is complex, comprising three major categories of transitions: openings/closings, preactivation, and desensitization. Interestingly, mutations at, e.g., the ligand binding site affect not only binding but often also more than one gating category, suggesting that structural determinants for distinct conformational transitions are shared. Gielen and co-workers (2015) proposed that the GABA_A_R desensitization gate is located at the second and third transmembrane segment. However, studies of our and others’ groups indicated that other parts of the GABA_A_R macromolecule might be involved in this process. In the present study, we asked how selected point mutations (β_2_G254V, α_1_G258V, α_1_L300V, and β_2_L296V) at the M2 and M3 transmembrane segments affect gating transitions of the α_1_β_2_γ_2_ GABA_A_R. Using high resolution macroscopic and single-channel recordings and analysis, we report that these substitutions, besides affecting desensitization, also profoundly altered openings/closings, having some minor effect on preactivation and agonist binding. Thus, the M2 and M3 segments primarily control late gating transitions of the receptor (desensitization, opening/closing), providing a further support for the concept of diffuse gating mechanisms for conformational transitions of GABA_A_R.

## Introduction

**Figure 1:**
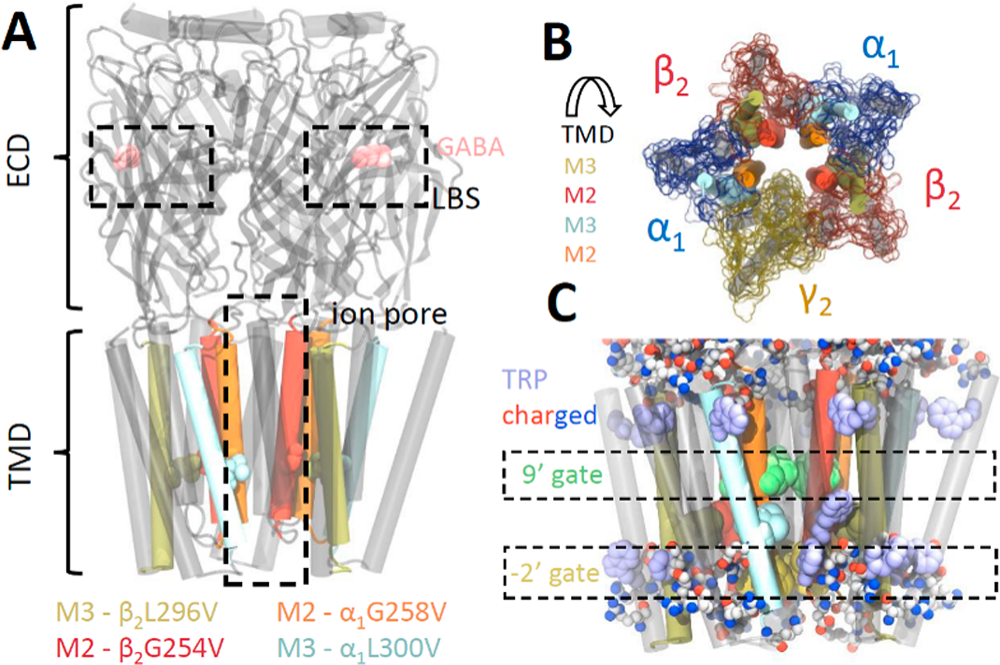
GABA_A_R structure and location of the β_2_G254, α_1_G258, α_1_L300, and β_2_L296 residues. (A) General structure of GABA_A_R with marked ECD and TMD domains, LBSs and ion pore. Respective α-helices (represented as cylinders) and investigated residues (in spherical representation) are marked with different colors. Notice the long distance between these residues and LBSs. (B) TMD seen from the ECD vestibule. Helices with investigated residues are marked as in part A, with other helices in gray; subunit surfaces are marked in respective colors. Notice the position of α-helices (M2s lining the pore, M3s in the “inner ring” of helices bundles). (C) Close look at TMD with marked two constriction regions: 9′ (β_2_Leu259, α_1_Leu264, and γ_2_Leu274, residues as green spheres) and −2′ (β_2_Ala248, α_1_Pro253, and γ_2_Pro263, residues as yellow spheres) gates. Related to anchoring in the lipid membrane, tryptophan residues are marked as violet spheres. All charged residues are presented in spherical representation with atoms colored according to charge (red = negative, blue = positive, gray = neutral).

GABA type A receptors (GABA_A_Rs) belong to the pentameric ligand-gated ion channel (pLGICs) family, together with, e.g., ionotropic serotonin receptor type 3, glycine receptor, or nicotinic acetylcholine receptor. GABA_A_Rs play a crucial role in inhibitory synaptic transmission in the adult mammalian brain.^1−3^ Dysfunction of GABAergic inhibition has been implicated in a number of neurological and psychiatric disorders such as epilepsy, autism, depression, and schizophrenia.^4−8^ Moreover, GABA_A_Rs are targets of numerous endogenous and exogenous modulators including, for instance, neurosteroids, benzodiazepines, anesthetics, and barbiturates.^9−13^ Recently, a major progress in determining the static structure of GABA_A_Rs has been achieved,^14−17^ although the precise molecular scenarios underlying various conformational transitions of this receptors remain unclear. A characteristic feature of pLGICs is that the activation process comprises vast portions of the macromolecules as the ligand binding site (LBS), located at extracellular domain (ECD), is positioned very far (approximately 50 Å)^2,18^ from the receptor gate, suggesting complexity of gating mechanisms (Figure 1A–C). Another dimension of GABA_A_R gating complexity is numerous conformational transitions which can be grouped in three main categories, openings/closings, preactivation, and desensitization, and each of them may be represented by transitions into more than one state (especially for openings/closings and desensitization, typically more than one state are identified).^19−23^

It is known that there are two possible constriction points/gates in the pore, one at the 9′ residue level, in the middle of the transmembrane domain (TMD), and the second one at the bottom, at the −2′ residue level (Figure 1C). According to structural data, the first one is responsible for channel opening/closing, whereas the second one is related to desensitization. Interestingly, mutations at the LBS are commonly found to affect not only binding but also gating, typically altering not just one gating category but two or three of them.^22,24−26^ This observation strongly suggests that structural determinants of the above-mentioned distinct gating transitions are not compartmentalized, but rather, specific structural elements of receptors might be shared upon different conformational transitions.

A particularly puzzling aspect with this respect is the process of desensitization. Gielen and co-workers^27^ proposed that the functioning of the GABA_A_R desensitization gate is regulated by interactions between the second and third transmembrane segments which are in close vicinity of the abovementioned −2′ residue, a presumed desensitization constriction site. However, studies from our group and others indicated involvement of other structural determinants in regulating the process of desensitization. We reported that a benzodiazepine, flurazepam, affected desensitization,^24,28^ and when we consider that the binding site for this modulator is located at ECD, very distantly from transmembrane segments indicated by Gielen et al.,^27^ it seems that desensitization might be also controlled by structures within ECD. Moreover, in our recent study,^22^ we provided evidence that mutation of the F45 residue, located close to the agonist binding site at the loop G of the α_1_ subunit, profoundly altered desensitization kinetics. Unexpectedly, we also found that mutation of the α_1_F14 and β_2_F31 residues, located “above” the orthosteric binding site in ECD, also had a strong impact on the desensitization transitions.^21^ Thus, our studies suggest that desensitization might structurally depend on vast fragments of the GABA_A_R macromolecule, raising a concept of a “diffuse” desensitization gating mechanism rather than its well defined localization. This means that, although the site of the final step of the desensitization transition is located at the constriction of the ion pore (at the level of −2′ residue in the TMD), the preceding structural rearrangements leading to this conformational change would comprise the vast parts of the macromolecule. Additionally, Germann and co-workers^29^ observed that propofol shifted the active-desensitized equilibrium toward the active states and attributed this finding to altered agonist affinities at these conformations, thus emphasizing the importance of long-range cross talk between GABA binding site, modulator binding site, and gates for opening and desensitization. To further address the issue of the structural determinants of desensitization transitions in GABA_A_R, we asked to what extent selected point mutations (β_2_G254V, α_1_G258V, α_1_L300V and β_2_L296V, Figure 1A–C) at the presumed desensitization gate proposed by Gielen et al.^27^ are specific for desensitization and to what extent they might also affect other conformational transitions of this receptor. Using high resolution macroscopic and single-channel recordings and analysis, we report that these substitutions, besides affecting desensitization, also profoundly altered openings/closings, having either no effect on preactivation or affecting it relatively weakly. Thus, the structure comprising the substituted residues appears to affect late gating transitions of the receptor, providing a further support for the concept of the diffuse gating mechanisms.

## Results and Discussion

### Analysis of Macroscopic Currents Mediated by the β_2_G254V, α_1_G258V, β_2_L296V, and α_1_L300V Mutants Indicate Diversified Effects on the Receptor Gating

**Figure 2:**
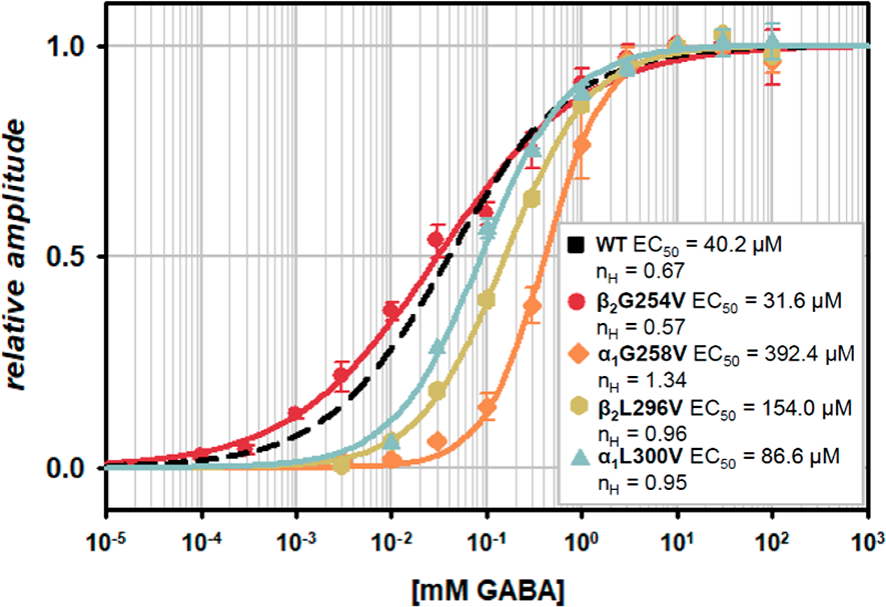
Mutations cause minor effects on agonist potency relative to WT.

We started to investigate the impact of the considered mutations at the bottom TMD area (M2 and M3 segments) on the receptor functions from assessment of agonist potency by analyzing the dose–response relationships (Figure 2). There was a slight reduction of EC_50_ value for the β_2_G254V mutant with respect to the WT receptor (31.6 vs 40.2 μM); however, this change is deemed insignificant, as the respective dose–response relationships nearly overlapped. For other mutants, EC_50_ value increased in ascending order for α_1_L300V, β_2_L296V and α_1_G258V (Figure 2, inset). The largest change was for the α_1_G258V mutant (392.4 μM) which was also accompanied by the largest alteration in the Hill coefficient (1.34 vs 0.63). Thus, in the case of agonist potency, relatively minor changes were observed, and the saturating GABA concentration for all of the mutants (and WT) was set at 10 mM. These trends in dose–response relationships suggest that the considered mutations might have a relatively minor impact on the agonist binding step.

Dose–response relationships normalized to current amplitudes elicited by saturating GABA concentrations (10 mM for all of the mutants and the WT receptors) that were fitted with the Hill equation (eq 1, Materials and Methods). Black dashed line represents fitting with the Hill equation (eq 1) of the dose–response relationship obtained by Brodzki et al.^30^ in the same experimental conditions. In the inset, EC_50_ values and Hill coefficients (*n*_H_) for the respective mutants and the WT receptors as the control are presented.

**Figure 3:**
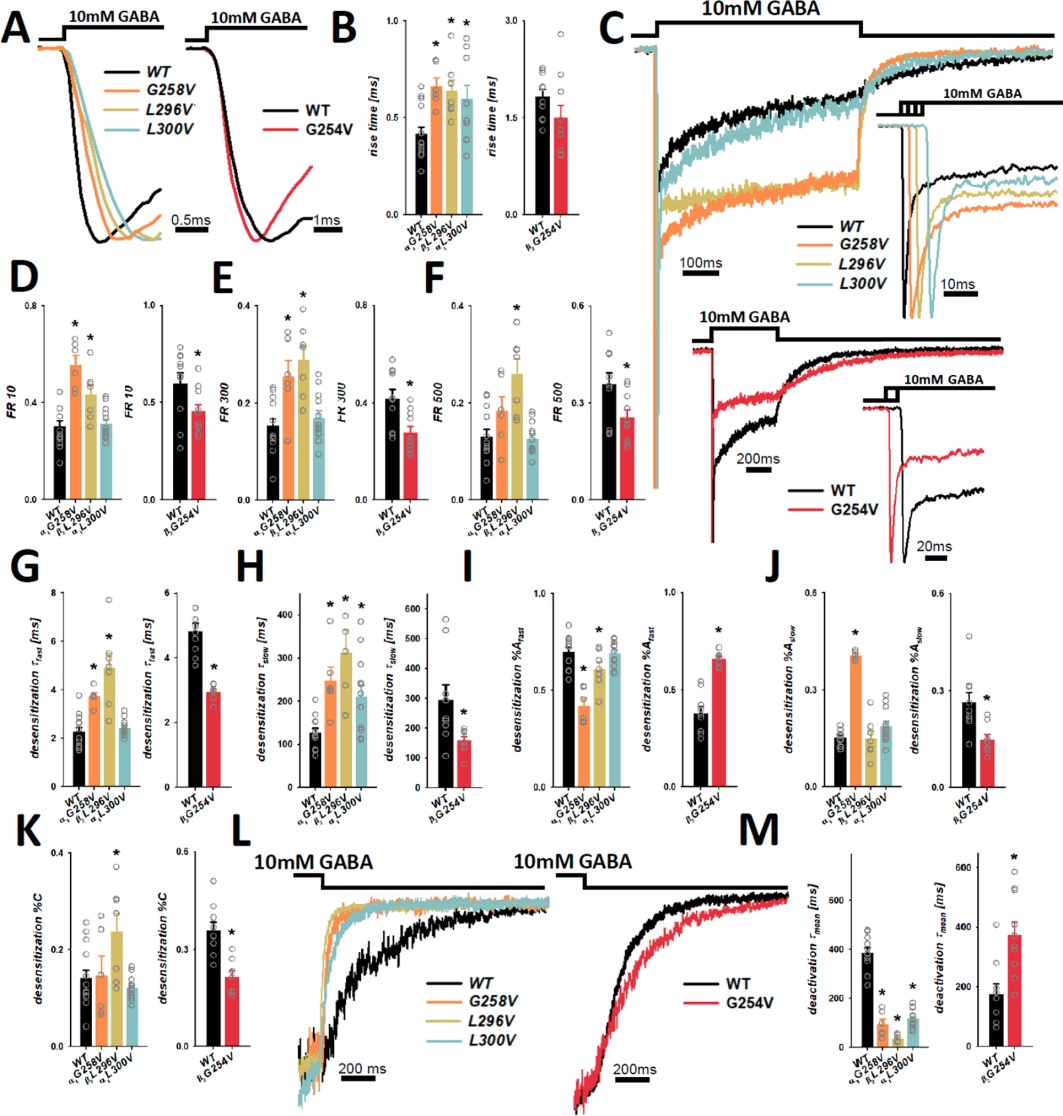
Kinetics of macroscopic currents evoked by saturating [GABA] applications is altered by the β_2_G254V, α_1_G258V, α_1_L300V and β_2_L296V mutations. (A) Typical normalized current traces of the onset phase, recorded from excised-patches, showing changes induced by the mutations in comparison to the WT receptors. Recordings presented for β_2_G254V with respective control (right panel) were obtained in the whole-cell configuration (lifted-cell). (B) Statistics for rise time values. (C) Typical normalized current traces evoked by 500 ms saturating GABA application or the initial 80–100 ms (right panel), showing differences in the time course and extent of fast and slow components of macroscopic desensitization for the α_1_G258V, α_1_L300V, β_2_L296V and β_2_G254V mutants compared to the WT receptors. (D–F) Statistics for FR10, F300, and FR500 values. (G, H) Statistics for time constants of fast (*τ*_fast_) and slow (*τ*_slow_) macroscopic desensitization components. (I, J) Statistics for percentages of the aforementioned desensitization components. (K) Statistics for the percentage (%*C*) of the stationary nondesensitizing current phase. (L) Typical normalized current traces representing effects of the α_1_G258V, α_1_L300V and β_2_L296V mutations (left traces) and the β_2_G254V mutant (right traces) on deactivation kinetics. (M) Statistics for mean deactivation time constant (τ_mean_). The insets above the current traces represent agonist application. Statistically significant differences between the mutants and WT are marked with asterisks. For absolute values see Table 1.

Next, to check for the impact of the considered mutations on receptor gating, current responses elicited by saturating [GABA] (10 mM) were examined. As already mentioned in the Materials and Methods, for WT and all of the mutants except for β_2_G254V, current traces were recorded from excised-patches, assuring the highest time resolution (Figure 3). For β_2_G254V, recordings were carried out in the whole-cell configuration (lifted-cell mode) due to poor expression and were compared to recordings of currents mediated by the WT receptors in the same configuration (Figure 3). For each current trace, the following parameters were determined, rise time (RT); desensitization = FR10, FR300, FR500, τ_fast_, %*A*_fast_, τ_slow_, %*A*_slow_, %*C* and deactivation = τ_mean_ (eqs 2 and 3, Materials and Methods), and the absolute values for all these parameters are presented in Table 1. The mean rise time of current responses was significantly slower in the case of the α_1_G258V, α_1_L300V and β_2_L296V receptors compared to WT (Figure 3A,B; Table 1). There was a slight trend to accelerate the current onset in the β_2_G254V mutant; however, it was not significant, and importantly, recordings for this receptor were made in the lifted-cell mode in which solution exchange is markedly slower than in the case of excised-patches (compare RT for WT: 0.41 and 1.83 ms in excised-patch and lifted-cell, respectively; Figure 3A,B; Table 1).

**Table 1:** Absolute Values Calculated for Kinetic Parameters in Macroscopic Current Analysis^a^

kinetic parameter	WT (EXP) *n* = 14	α_1_ G258V *n* = 6	β_2_ L296V *n* = 7	α_1_ L300V *n* = 12	WT (LC) *n* = 10	β_2_ G254V *n* = 10
rise time [ms]	0.41 ± 0.04	0.66 ± 0.04*	0.64 ± 0.06*	0.60 ± 0.07*	1.83 ± 0.19	1.50 ± 0.11
		***p* < 0.001**	***p* = 0.003**	***p* = 0.037**		
FR 10	0.30 ± 0.02	0.56 ± 0.04*	0.43 ± 0.04*	0.31 ± 0.02	0.60 ± 0.06	0.45 ± 0.04*
		***p*< 0.001**	***p* = 0.005**			***p* = 0.047**
FR 300	0.15 ± 0.02	0.26 ± 0.03*	0.29 ± 0.03*	0.17 ± 0.01	0.42 ± 0.04	0.28 ± 0.03*
		***p* = 0.005**	***p* < 0.001**			***p* < 0.001**
FR 500	0.13 ± 0.06	0.18 ± 0.03	0.26 ± 0.03*	0.13 ± 0.01	0.36 ± 0.04	0.26 ± 0.02*
			***p* < 0.001**			***p* = 0.030**
*τ*_fast_ [ms]	2.27 ± 0.19	3.71 ± 0.18*	4.90 ± 0.61*	2.42 ± 0.10	4.82 ± 0.26	2.94 ± 0.10*
		***p* < 0.001**	***p* < 0.001**			***p* = 0.001**
*τ*_slow_ [ms]	127.98 ± 9.92	247.19 ± 31.88*	313.09 ± 51.82*	210 ± 26.47*	294.69 ± 51.77	158.48 ± 14.34*
		***p* < 0.001**	***p* = 0.001**	***p* = 0.021**		***p* = 0.018**
%*A*_fast_	0.70 ± 0.02	0.42 ± 0.04*	0.61 ± 0.04*	0.69 ± 0.02	0.38 ± 0.10	0.64 ± 0.07*
		***p* < 0.001**	***p* = 0.042**			***p* = 0.007**
%*A*_slow_	0.15 ± 0.03	0.41 ± 0.01*	0.15 ± 0.02	0.19 ± 0.02*	0.26 ± 0.03	0.15 ± 0.02*
		***p* < 0.001**		***p* = 0.041**		***p* = 0.007**
%*C*	0.14 ± 0.02	0.15 ± 0.04	0.24 ± 0.04*	0.12 ± 0.01	0.36 ± 0.03	0.22 ± 0.02*
			***p* = 0.013**			***p* < 0.001**
deactivation *τ*_mean_ [ms]	383.87 ± 89.49	93.40 ± 21.36*	36.03 ± 5.91*	116.50 ± 10.60*	173.36 ± 37.31	375.42 ± 43.17*
		***p* < 0.001**	***p* < 0.001**	***p* < 0.001**		***p* = 0.003**

a Significant changes relative to the respective WT control (EXP = excised-patch or LC = lifted-cell mode) are marked in bold with an asterisk (*), and the corresponding *p* value is disclosed; *n* = number of patches.

Because the mutations are located at the presumed desensitization gate,^27^ a particular care was taken in our analysis to characterize the macroscopic desensitization (hence numerous parameters describing this process in Table 1). The fraction of nondesensitized current was measured at 10, 300 and 500 ms after the peak (FR10, FR300 and FR500). For the α_1_G258V and β_2_L296V mutants, FR10 and FR300 were significantly increased (for β_2_L296V also FR500) with respect to WT, indicating the decreased extent of desensitization (Figure 3C,E–G). Moreover, in these two mutated receptors, both fast and slow desensitization time constants, τ_fast_ and τ_slow_, were slowed down, while percentages of the fast components, %*A*_fast_, were reduced compared to those of WT (%*A*_slow_ increased only in α_1_G258V, Figure 3C,G–J; Table 1). Additionally, the stationary fraction of current (%*C*) was significantly increased for the β_2_L296V mutant (Figure 3K; Table 1). Thus, the α_1_G258V and β_2_L296V mutations slowed down the time course and reduced the extent of macroscopic desensitization (at least at some time points). In the case of the α_1_L300V mutation, we observed an increased value of the slow desensitization time constant, τ_slow_, with a minor (although significant) increase in percentage (%*A*_slow_) of this component (Figure 3C,G–J; Table 1). We also analyzed desensitization characteristics for the β_2_G254V mutant for which, as already mentioned, recordings were made in the lifted-cell mode. Contrary to the previously analyzed mutants, for the β_2_G254V receptors, both the rate and extent of macroscopic desensitization were upregulated, although this effect was relatively small. Indeed, FR10, FR300 and FR500 were reduced, and fast and slow time constants τ_fast_ and τ_slow_ were accelerated (with %*A*_fast_ increased and %*A*_slow_ reduced); the stationary fraction of current (%*C*) was significantly reduced (Figure 3C–K; Table 1). We additionally analyzed the time course of deactivation (assessed as τ_mean_, see Materials and Methods) which was significantly accelerated for the α_1_G258V, β_2_L296V and β_2_L300V receptors, but for the mutation enhancing desensitization, β_2_G254V, deactivation was slowed down with respect to WT (Figure 3L,M; Table 1).

### Kinetic Modeling of Macroscopic Data

In order to provide detailed information regarding the impact of the considered mutations on receptor gating, model simulations were carried out using the optimization routine of ChannelLab software (see Materials and Methods). As pointed out by Colquhoun and Lape,^31^ in general, macroscopic modeling is known to be susceptible to model overparametrization which means that, in practice, it is hard to obtain stable and reproducible fits for complex models. For this reason, we carried out the macroscopic modeling for simplified schemes (simpler than in the case of single-channel data, see below).

**Figure 4:**
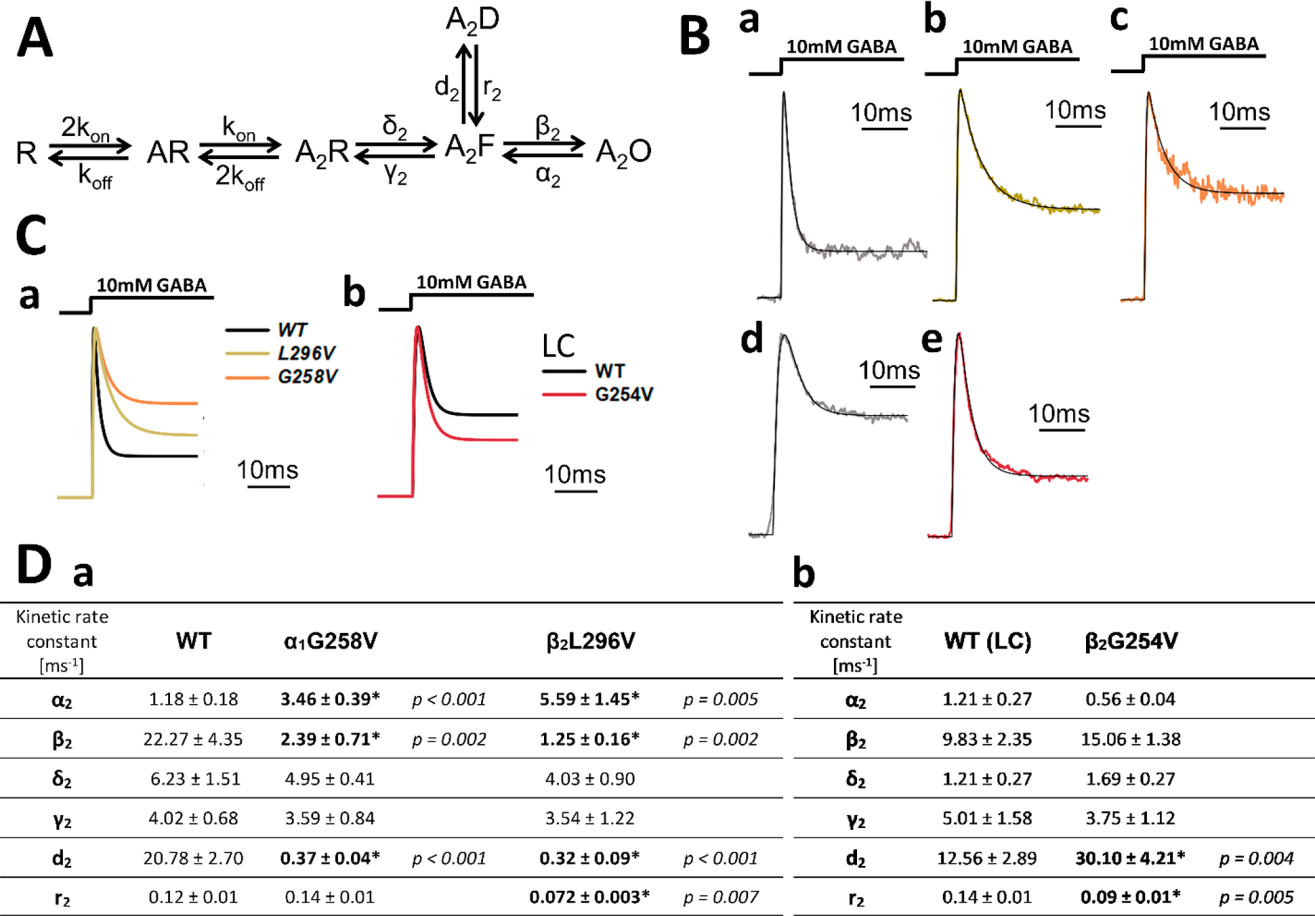
Macroscopic modeling indicates that β_2_L296V and α_1_G258V affect the receptor gating differently than the β_2_G254V mutation does. (A) Scheme of a kinetic model (fJWM from Szczot et al.^26^). In the scheme: R = unbound receptor; AR = singly bound receptor; A_2_R = doubly bound receptor; A_2_F = flipped state; A_2_O = open state; A_2_D = desensitized state. For all of the mutants, this model was used. (B) Exemplary model fits for recordings carried out in the excised-patch configuration for (a) WT receptors, (b) β_2_L296V, (c) α_1_G258V, (d) WT in the lifted-cell mode, and (e) β_2_G254V in the same mode. To improve the visibility, current traces for the WT receptors are drawn with a light gray line instead of a black one, as in part Ca,b. (C) (a, b) Normalized simulated traces for WT and the mutants generated for the mean values of the rate constants presented in parts Da and De, respectively, in the ∼30 ms time window. Traces shown in part b were modeled for recordings from the lifted cells. (D) (a, b) Results of modeling presented in the table as mean values of the kinetic rate constants for each gating transition. Significant changes in the rate constants vs WT are marked in bold with an asterisk (*), and the corresponding *p* value is disclosed. In part b, presented rates are for the lifted-cell mode recordings.

In the case of the α_1_G258V and β_2_L296V mutations, the major manifestations of the altered kinetic phenotype with respect to the WT receptors (slower onset and reduced rate and extent of rapid desensitization) could be observed within the time window of approximately 30 ms. We thus restricted our analysis for this time window and fitted the respective traces (for WT and the mutants) with a simplified model with one open and one (rapid) desensitized state (Figure 4A, exemplary fits in part Ba–c, simulated traces in part Ca). Using this approach, the values of the rate constants for the WT receptors (Figure 4Da) were comparable to those obtained in our previous studies^24,26^ and the kinetic features of current responses mediated by these receptors were well-reproduced by curves obtained from modeling within the considered time range (data not shown). Also, in the case of the considered mutants, fitting with the simplified model to approximately 30 ms intervals of current responses well-reproduced the time course of recorded currents. As expected from observations on macroscopic desensitization (Table 1), for both α_1_G258V and β_2_L296V mutations, model fitting revealed a large and significant reduction of the desensitization rate *d*_2_, and the resensitization *r*_2_ parameter was significantly decreased for β_2_L296V but was unaltered for the α_1_G258V mutant (Figure 4Da). However, besides changes in the microscopic desensitization (*d*_2_ and *r*_2_), these mutations also strongly affected the opening/closing transitions by dramatically reducing the opening β_2_ and by increasing the closing α_2_ rates (Figure 4Da). The parameters that characterize preactivation (δ_2_ and γ_2_) tended to be reduced in the case of both mutants, but these changes were not statistically significant. In Figure 4Ca, we show normalized simulated traces for WT and the mutants generated for the mean values of the rate constants presented in Figure 4Da, for the 30 ms time window. Taken altogether, the α_1_G258V and β_2_L296V mutations showed a clear trend to impair late gating transitions (opening/closing and desensitization), having little or no effect on binding and preactivation.

**Figure 5:**
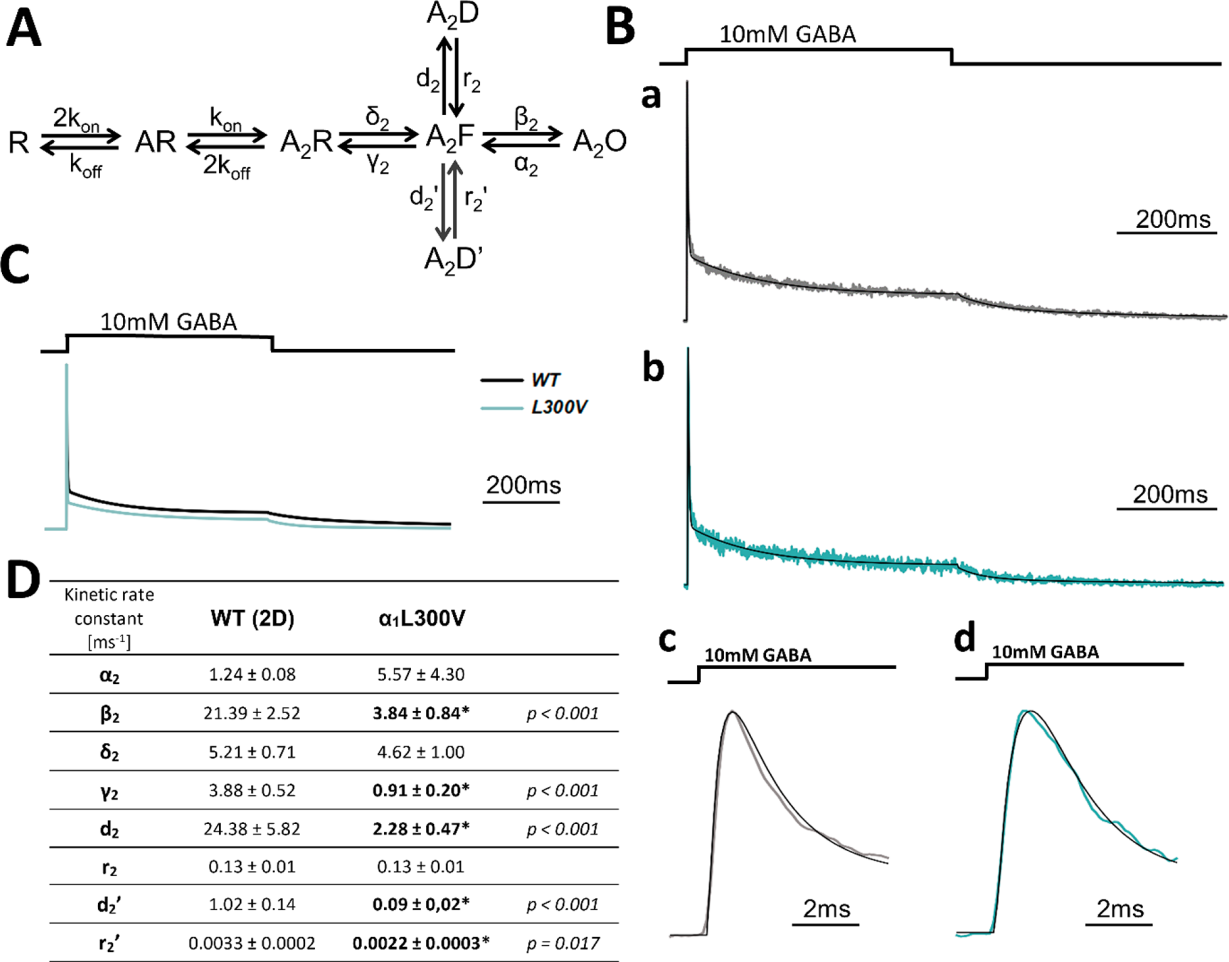
Macroscopic modeling indicates that the α_1_L300V mutation primarily affects the desensitization and opening rates. (A) Scheme of a kinetic model (fJWM from Szczot et al.^26^) with additional desensitized state. In the scheme: R = unbound receptor; AR = singly bound receptor; A_2_R = doubly bound receptor; A_2_F = flipped state; A_2_O = open state; A_2_D and A_2_D′ = desensitized states. This model was used for the α_1_L300V mutant. (B) Exemplary model fits for recordings in the excised-patch configuration for (a) WT receptor and (b) α_1_L300V (c) full duration pulse and (d) zoomed on the onset phase. To improve visibility, current traces for the WT receptors are drawn with a light gray line instead of a black one, as in part C. (C) Normalized simulated traces for WT and the α_1_L300V mutant generated for the mean values of the rate constants presented in part D in the ∼1000 ms time window. (D) Results of modeling presented in the same manner as in that in Figure 4D, including additional *d*_2_′ and *r*_2_′ rates.

The kinetic phenotype of currents mediated by the β_2_L300V mutant was qualitatively different from that described above for α_1_G258V and β_2_L296V. All the kinetic features related to desensitization were not different from those determined for the WT receptors except for the time constant and percentage of the slow component (τ_slow_ and %*A*_slow_, Table 1). In this situation, the strategy to implement the simplified model (Figure 4A) to fit the time course of responses within a limited time window (as in the case of the α_1_G258V and β_2_L296V mutations) could not be applied. Thus, the extended model with two desensitized states had to be used (Figure 5A). In the fitting with the extended model, we considered the entire trace of current mediated by the α_1_L300V mutant with the slow desensitization phase and deactivation which also differed from that in the WT receptor mediated currents. Fitting of the entire trace with the extended model was somehow problematic, especially when starting the optimization procedure with initial guessing values for the rate constants distant from the optimized ones. Finally, it was possible to find a set of the optimized rate constants for each considered trace (exemplary fits for WT and the mutant in Figure 5Ba–d), but the initial guesses had to be set very close to the optimized values, otherwise either no convergence was observed or a nonreproducible variable and often extreme values of the rate constants were obtained. Although, at the end, we managed to collect the statistics for the model fitting for this mutant (see Figure 5C for simulated traces), but we judge this set of fits as relatively poor, even if each individual fit fairly reproduced the time course of the current response. As shown in Figure 5D, in the α_1_L300V mutant, the three desensitization rates *d*_2_, *d*_2_′, and *r*_2_′ were significantly reduced compared to those of the WT receptors, but the *r*_2_ rate was not affected by the mutation. Similar to those of the α_1_G258V and β_2_L296V mutations, the opening rate β_2_ was strongly reduced in the mutant, but the closing rate α_2_ was not significantly affected (note particularly large scatter for this rate constant). Finally, the preactivation (flipping) rate δ_2_ was unaffected in β_2_L300V, but contrary to the α_1_G258V and β_2_L296V mutants, the unflipping rate γ_2_ was significantly reduced. In Figure 5C, we show simulated current responses for WT and the β_2_L300V mutant for the mean values of the rate constants presented in Figure 5D.

Because of low activity, for the β_2_G254V mutant, current responses were collected in the whole-cell configuration, and for the sake of comparison, controls for the WT receptors were recorded in the same conditions; a new modeling was done for these data. The rates for WT and the mutant were estimated in the same manner as that for α_1_G258V and β_2_L296V, using the model with a single desensitized state (Figure 4A) and for the ∼30 ms fitting time window (see Figure 4Bd,e). In these conditions, the temporal resolution is markedly smaller than that for recordings from excised-patches, and this might explain a large data scatter for the rate constants estimated in modeling (Figure 4Db). Nevertheless, the models could be accurately fitted to experimental traces, allowing to assess the impact of this mutation on conformational transitions. The only significant changes were found for *d*_2_ (increased) and *r*_2_ (decreased), in agreement with generally stronger macroscopic desensitization observed in this mutant. Changes of other rates were not statistically significant. Figure 4Cb shows simulated current responses for WT and the β_2_G254V mutant for the mean values of the rate constants presented in Figure 4Db.

### Single-Channel Activity Reveals Major Changes in Gating Properties of the α_1_G258V, α_1_L300V, and β_2_L296V Mutated Receptors

**Figure 6:**
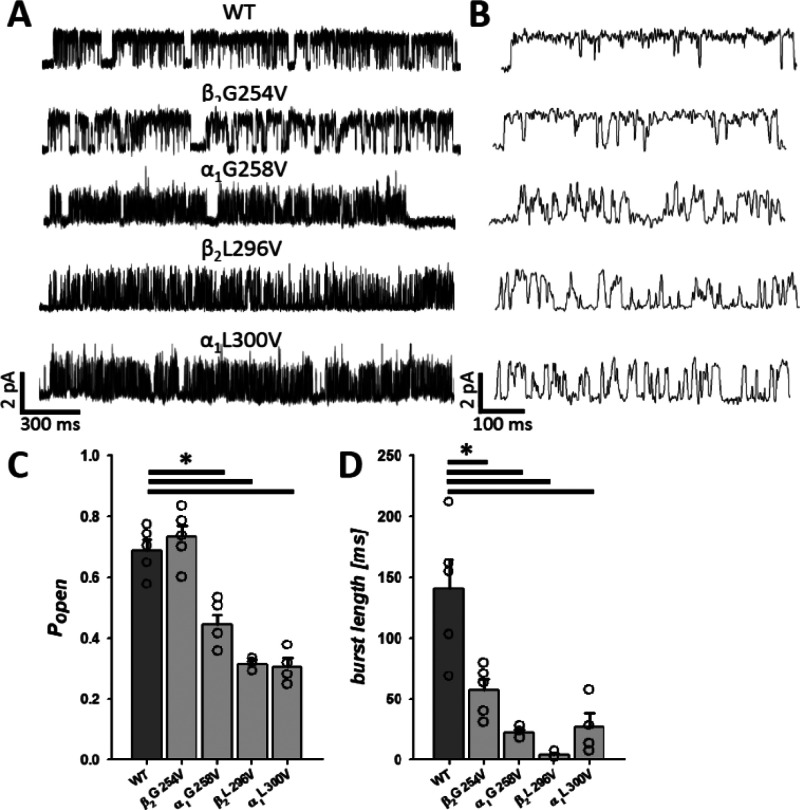
Single-channel activity reveals diversified effects of mutations on activity patterns, burst lengths, and open probabilities. (A) Examples of typical clusters of single-channel activity (the predominant mode, see Materials and Methods) for the respective mutants and the WT receptors, showing marked changes in patterns of channel opening and closing. (B) Same as in part A but in expanded time scale. (C) Statistics for mean open probability (*P*_open_) calculated for bursts. (D) Statistics for the mean burst length. Note that these values are significantly shortened with respect to the WT receptors for all of the mutants.

To more precisely investigate effects of the mutations on the GABA_A_R activity, especially taking into account that modeling of macroscopic data was not particularly precise for some of the mutants, the analysis of single-channel currents elicited by saturating [GABA] (10 mM GABA) was performed. This analysis was focused on the predominant mode of activity for each considered mutant (see Materials and Methods). The difference between single-channel activity mediated by the α_1_G258V, α_1_L300V and β_2_L296V mutants and the WT receptors was immediately apparent when just looking at the traces (Figure 6A,B). However, in the case of β_2_G254V, the phenotype of activity similar to that of the WT receptors could be noticed (Figure 6A,B). Burst analysis (see Materials and Methods) revealed that, for each considered mutant, except for β_2_G254V, a strong reduction in the open probability (*P*_open_) took place (*P*_open_ for WT: 0.69 ± 0.04, *n* = 5; for β_2_G254V: 0.73 ± 0.04, *n* = 5; for α_1_G258V: 0.45 ± 0.03, *n* = 5, *p* < 0.001; for β_2_L296V: 0.32 ± 0.01, *n* = 3, *p* < 0.001; for α_1_L300V: 0.31 ± 0.03, *n* = 4, *p* < 0.001; Figure 6C). However, all of the mutations, including β_2_G254V, caused a large reduction of the mean burst length (for WT: 140.29 ± 24.78 ms, *n* = 5; for β_2_G254V: 57.32 ± 9.27 ms, *n* = 5, *p* = 0.014; for α_1_G258V: 22.27 ± 2.41 ms, *n* = 4, *p* = 0.016; for β_2_L296V: 4.24 ± 1.82 ms, *n* = 3, *p* = 0.006; for α_1_L300V: 27.06 ± 11.27 ms, *n* = 4, *p* = 0.007; Figure 6D).

**Figure 7:**
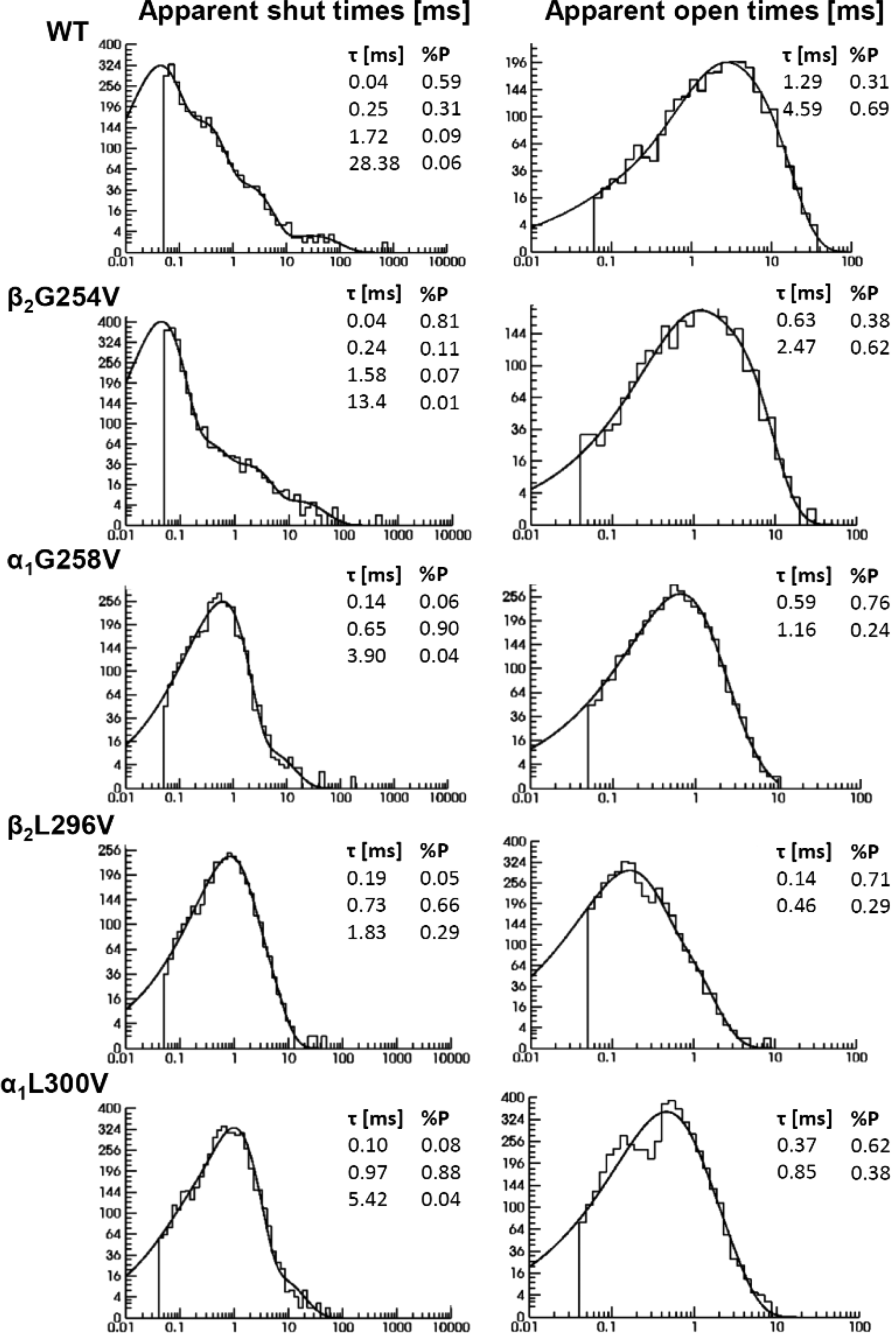
Distributions of apparent shut and open times for WT and the β_2_G254V, α_1_G258V, α_1_L300V and β_2_L296V mutants indicate major changes in gating properties. Examples of typical dwell time distributions for shut times (left panel) and open times (right panel) that were fitted with probability density functions and the respective parameters of each component (time constant τ and its percentage %*P*) are presented in insets. In shut time distributions for WT and β_2_G254V, four components are present, while for the α_1_G258V, α_1_L300V and β_2_L296V mutants, there are only three. For statistics, see Table 2 (shut times) and Table 3 (open times).

Next, the dwell time distributions for closings and openings were analyzed. Shut time distributions for the WT receptors consisted of four components (at 40–80 μs resolution), and the same number of components was found for β_2_G254V. For all of the remaining mutants, only three components were needed. In the case of open times, two components (at 30–90 μs resolution) were consistently detected in all of the considered cases. Typical examples of fitted distributions for shut and open times are shown in Figure 7. The statistics for shut time distributions are presented in Table 2. Because in the case of the α_1_G258V, β_2_L296V and α_1_L300V mutations only three shut times components were present, the comparison to WT was done for the three shortest components. This strategy seems justified, as the time constant of the slowest, fourth component for the WT receptors was by far larger than any of those in the mutants.

**Table 2:** Mean Values of Parameters of Shut Time Distributions

shut time	τ_1_ [ms]	%*P*_1_	τ_2_ [ms]	%*P*_2_	τ_3_ [ms]	%*P*_3_	τ_4_ [ms]	%*P*_4_
WT *n* = 5	0.04 ± 0.003	0.57 ± 0.03	0.26 ± 0.01	0.32 ± 0.02	1.55 ± 0.11	0.10 ± 0.01	20.42 ± 2.71	0.01 ± 0.002
	(0.06 ± 0.01)	(0.45 ± 0.05)	(0.34 ± 0.02)	(0.38 ± 0.05)	(1.70 ± 0.17)	(0.16 ± 0.02)	(20.95 ± 3.03)	(0.01 ± 0.002)
	[0.05 ± 0.01]	[0.51 ± 0.03]	[0.32 ± 0.02]	[0.35 ± 0.04]	[1.68 ± 0.17]	[0.13 ± 0.02]	[20.94 ± 3.03]	[0.01 ± 0.001]
β_2_ G254V *n* = 5	0.05 ± 0.01	0.68 ± 0.05	0.36 ± 0.05	0.17 ± 0.02*	2.26 ± 0.28*	0.10 ± 0.02	19.25 ± 3.70	0.04 ± 0.01*
				***p* < 0.001**	***p* = 0.043**			***p* = 0.038**
	(0.06 ± 0.01)	(0.57 ± 0.04)	(0.50 ± 0.02)	(0.25 ± 0.03)	(2.63 ± 0.35)	(0.15 ± 0.03)	(18.35 ± 3.35)	(0.04 ± 0.02)
	[0.06 ± 0.01]	[0.61 ± 0.04]	[0.49 ± 0.02)	[0.23 ± 0.03]	[2.61 ± 0.35]	[0.13 ± 0.03]	[18.31 ± 3.85]	[0.03 ± 0.01]
α_1_ G258V *n* = 5	0.23 ± 0.04*	0.08 ± 0.02*	0.85 ± 0.06*	0.87 ± 0.01*	3.41 ± 0.32*	0.05 ± 0.01*		
	***p* = 0.008**	***p* < 0.001**	***p* = 0.008**	***p* < 0.001**	***p* < 0.001**	***p* = 0.038**		
	(0.21 ± 0.05)	(0.12 ± 0.04)	(0.89 ± 0.07)	(0.83 ± 0.03)	(3.84 ± 0.88)	(0.05 ± 0.01)		
	[0.20 ± 0.05]	[0.17 ± 0.05]	[0.76 ± 0.07)	[0.79 ± 0.04]	[3.82 ± 0.81]	[0.04 ± 0.01]		
β_2_ L296V *n* = 3	0.19 ± 0.03*	0.08 ± 0.04*	0.88 ± 0.12*	0.69 ± 0.07*	2.24 ± 0.32*	0.22 ± 0.04*		
	***p* = 0.036**	***p* < 0.001**	***p* < 0.001**	***p* < 0.001**	***p* = 0.043**	***p* = 0.015**		
	(0.10 ± 0.01)	(0.10 ± 0.02)	(0.96 ± 0.16)	[0.74 ± 0.06)	[2.61 ± 0.42)	(0.16 ± 0.07)		
	[0.09 ± 0.01]	[0.16 ± 0.02]	[0.73 ± 0.11)	[0.76 ± 0.05]	[2.51 ± 0.44]	[0.09 ± 0.03]		
α_1_ L300V *n* = 4	0.11 ± 0.02*	0.05 ± 0.01*	1.08 ± 0.05*	0.87 ± 0.03*	4.75 ± 0.83*	0.08 ± 0.03		
	***p* = 0.005**	***p* < 0.001**	***p* = 0.016**	***p* = 0.036**	***p* = 0.016**			
	(0.11 ± 0.005)	(0.06 ± 0.02)	(1.07 ± 0.05)	(0.85 ± 0.04)	(4.22 ± 0.58)	(0.09 ± 0.03)		
	[0.11 ± 0.004]	[0.08 ± 0.02]	[0.86 ± 0.03)	[0.86 ± 0.03)	[4.16 ± 0.60]	[0.05 ± 0.01]		

As expected from the appearance of the single-channel traces and the exemplary shut time distribution (Figures 6 and 7), the impact of the β_2_G254V mutation was rather weak: the only difference compared to WT was a decrease in the percentage of the second component (%*P*_2_, Table 2). However, the α_1_G258V, β_2_L296V and α_1_L300V mutations profoundly altered the shut time distributions by not only affecting the number of components but also by dramatically altering the respective parameters. Most notably, typical shut time distributions for these mutants (Figure 7) showed a dramatic reduction in the bin content for the short-lasting closures. Indeed, in the case of these mutants, the shortest time constant (τ_1_) is markedly increased and its percentage (%*P*_1_) is strongly reduced. Moreover, in the case of the second component, both the time constant (τ_2_) and its percentage (%*P*_2_) are markedly increased. These alterations in the two shortest shut time components reveal an overall robust increase in the short closure duration, indicative for a strong reduction of the opening rate of the receptor. Moreover, in the case of β_2_L296V, the percentage of the third component (%*P*_3_) was slightly increased.

Time constants and percentages for the components present in the distributions for the WT receptors and β_2_G254V, α_1_G258V, α_1_L300V and β_2_L296V mutants: without brackets = experimental parameters; normal brackets = simulated with experimental resolution; square brackets = simulated with 0 μs resolution. Statistically significant changes relative to WT are highlighted in bold with an asterisk (*) and the corresponding *p* value; *n* = number of patches.

To complete our analysis of dwell time distributions, open times were investigated (Figure 7; Table 3). The β_2_G254V mutation did not affect the open times significantly, which further confirmed its similarity to the WT phenotype. However, in the rest of the mutants, a clear trend of shortening of opening was observed. Indeed, in α_1_G258V, the percentage of the fastest component (%*P*_1_) was significantly increased (with concomitant decrease in percentage of slower component %*P*_2_), whereas in the case of β_2_L296V and α_1_L300V, both open time constants (τ_1_ and τ_2_) were significantly shortened (Table 3). Based on the distribution parameters, mean (weighted) open times (%*P*_1_τ_1_ + %*P*_2_τ_2_) were calculated, and for the α_1_G258V, α_1_L300V and β_2_L296V mutants this value was significantly reduced compared to that of WT (Table 3). Notably, in the case of β_2_L296V, mean open time was shortened by nearly 1 order of magnitude, indicating a particularly strong impact on the closing transition. Taken together, except for the β_2_G254V mutation, which did not affect single-channel openings in all the remaining mutants, an overall trend to shorten the open times was observed, suggesting an increased closing rate compared to that of the WT receptors.

**Table 3:** Mean Values of Parameters of Open Time Distribution

open time	τ_1_ [ms]	%*P*_1_	τ_2_ [ms]	%*P*_2_	mean open time [ms]
WT *n* = 5	1.02 ± 0.14	0.55 ± 0.08	2.86 ± 0.49	0.45 ± 0.08	1.92 ± 0.42
	(0.90 ± 0.10)	(0.87 ± 0.04)	(2.90 ± 0.56)	(0.13 ± 0.04)	(1.16 ± 0.17)
	[0.57 ± 0.05]	[0.94 ± 0.02]	[2.77 ± 0.53]	[0.06 ± 0.02]	[0.69 ± 0.08]
β_2_G254V *n* = 5	0.88 ± 0.26	0.42 ± 0.06	3.06 ± 0.82	0.58 ± 0.07	2.22 ± 0.60
	(0.99 ± 0.21)	(0.81 ± 0.04)	(3.84 ± 0.62)	(0.19 ± 0.04)	(1.51 ± 0.23)
	[0.64 ± 0.14]	[0.8 ± 0.04]	[3.62 ± 0.61]	[0.11 ± 0.04]	[0.95 ± 0.16]
α_1_G258V *n* = 5	0.55 ± 0.09*	0.82 ± 0.04*	1.39 ± 0.28*	0.18 ± 0.04*	0.68 ± 0.10*
	***p* = 0.019**	***p* = 0.016**	***p* = 0.031**	***p* = 0.016**	***p* = 0.008**
	(0.47 ± 0.09)	(0.80 ± 0.03)	(1.22 ± 0.30)	(0.20 ± 0.03)	(0.60 ± 0.10)
	[0.42 ± 0.09]	[0.84 ± 0.02]	[1.19 ± 0.30]	[0.16 ± 0.02]	[0.58 ± 0.14]
β_2_L296V *n* = 3	0.12 ± 0.001*	0.57 ± 0.03	0.52 ± 0.04*	0.42 ± 0.04	0.26 ± 0.03*
	***p* = 0.003**		***p* = 0.011**		***p* = 0.036**
	(0.12 ± 0.01)	(0.62 ± 0.05)	(0.47 ± 0.04)	(0.33 ± 0.05)	(0.24 ± 0.03)
	[0.11 ± 0.01]	[0.72 ± 0.05]	[0.45 ± 0.04]	[0.28 ± 0.05]	[0.21 ± 0.03]
α_1_L300V *n* = 4	0.20 ± 0.07*	0.52 ± 0.12	0.62 ± 0.11*	0.48 ± 0.12	0.39 ± 0.06*
	***p* = 0.002**		***p* = 0.005**		***p* = 0.016**
	(0.20 ± 0.07)	(0.56 ± 0.12)	(0.61 ± 0.12)	(0.44 ± 0.12)	(0.36 ± 0.05)
	[0.19 ± 0.06]	[0.60 ± 0.12]	[0.59 ± 0.12]	[0.40 ± 0.12]	[0.33 ± 0.05]

Time constants and the percentages of the two open time components and mean open time for the WT, β_2_G254V, α_1_G258V, α_1_L300V and β_2_L296V receptors: without brackets = experimental parameters; normal brackets = simulated with experimental resolution; square brackets = simulated with 0 μs resolution. Statistically significant changes relative to WT are highlighted in bold with an asterisk (*) and the corresponding *p* value; *n* = number of patches.

### Kinetic Modeling of Single-Channel Data

**Figure 8:**
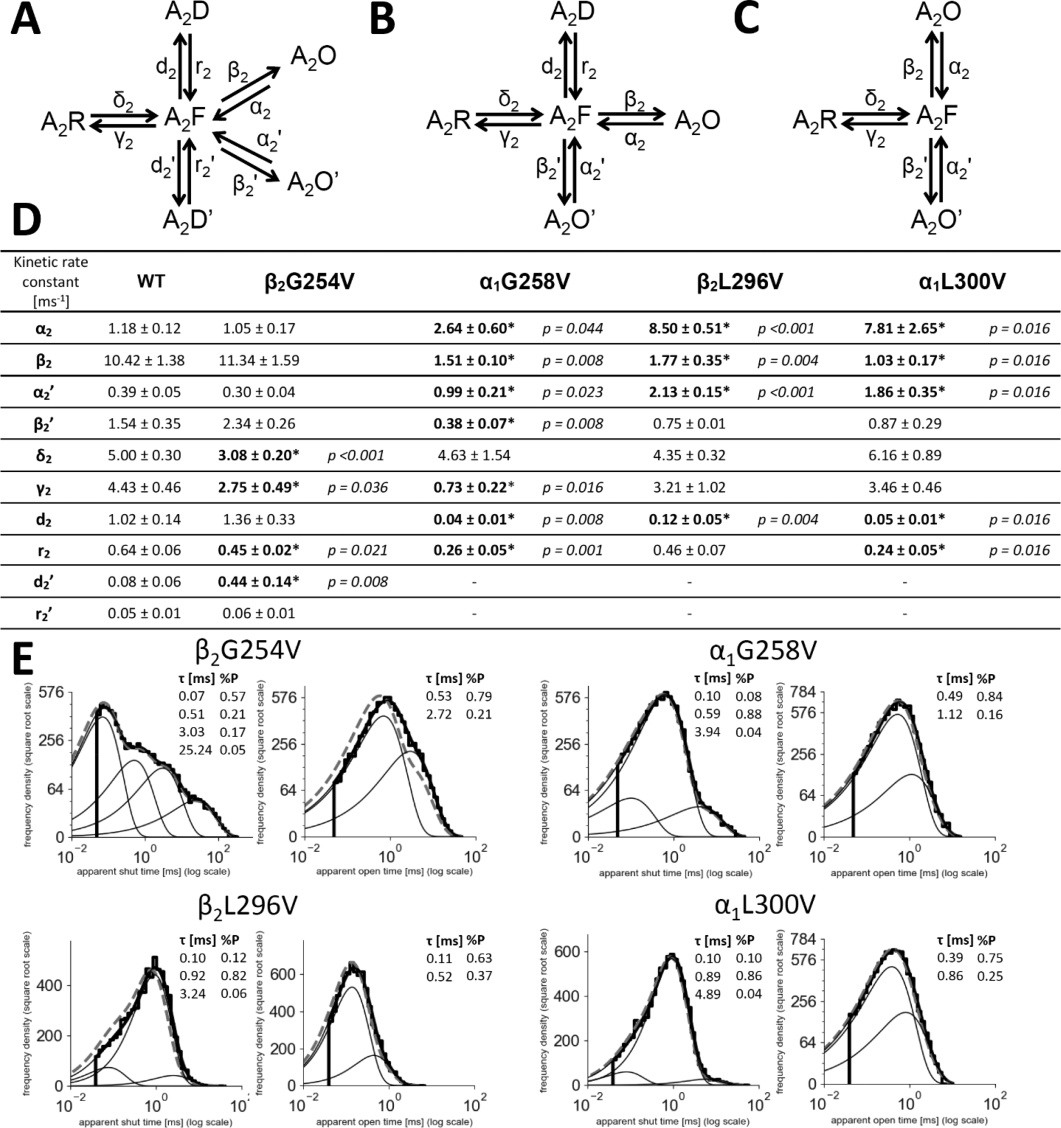
Results of the single-channel modeling reveal major changes caused by the α_1_G258V, α_1_L300V and β_2_L296V mutations on opening, closing and desensitization rates but only a minor effect on preactivation. (A) Scheme of the full kinetic model from Kisiel et al.^23^ with two open and two desensitized states that was used for modeling of the WT and β_2_G254V receptors. In the scheme: A_2_R = doubly bound receptor; A_2_F = flipped state; A_2_O and A_2_O′ = open states; A_2_D and A_2_D′ = desensitized states. (B) The same model as that in part A but with one desensitized state (A_2_D). This model was used for modeling of the mutants with only three shut time components (α_1_G258V, α_1_L300V and β_2_L296V). (C) A scheme of a kinetic model with only two closed (A_2_R and A_2_F) and two open (A_2_O and A_2_O′) states and no desensitization, which was used in the intermediate step of modeling for the α_1_G258V, α_1_L300V and β_2_L296V mutants (see single-channel modeling section). (D) Results of modeling presented in the table as mean values of the kinetic rate constants for each gating transition for the considered mutants. Significant changes in the rate constants vs WT are marked in bold with an asterisk (*), and the corresponding *p* value is disclosed. (E) Solid black lines present examples of the simulated dwell time distributions of the frequency density functions overlaid on the experimental shut and open time distributions (insets present time constants τ and percentages %*P* of the components present in the simulated distributions). Solid gray, thin lines outline the exponential components in apparent shut and open time distributions. Gray, thick, dashed lines show the distributions obtained with an application of the corrections for missed events.

Having detected single-channel events and characterized the dwell time distributions (Tables 2 and 3), we used HJCFIT software to perform modeling of the single-channel data. Because all of the receptors had two components in open time distributions, in all models, two open states were included. Also because the WT receptors and the β_2_G254V mutant had shut time distributions consisting of four components, the extended model from Kisiel et al.^23^ with four shut states (one closed, one preactivated, and two desensitized states) was employed (Figure 8A). Consistently, in the case of the α_1_G258V, α_1_L300V and β_2_L296V mutants, three shut states (one desensitized state only) were included in the model (Figure 8B). These model schemes for gating are more complex than the ones used in the macroscopic modeling (Figures 4 and 5), but as pointed out by Colquhoun and Lape,^31^ the single-channel modeling is less susceptible to model overparametrization than the macroscopic one. Clearly, because the recordings were carried out in the stationary conditions in the presence of saturating [GABA], the binding steps in the schemes were omitted.

Model fitting for the β_2_G254V mutant generally confirmed that its kinetic phenotype was similar to that of the WT receptors, although some relatively minor differences were found. Specifically, the flipping and unflipping rates δ_2_ and γ_2_, were slightly (but significantly) reduced and also, the rates describing desensitization (*r*_2_ and *d*_2_) were altered indicating enhancement of desensitization (Figure 8D) which corroborates our macroscopic findings.

Upon applying the optimization procedures to fit the selected models to the data collected for the α_1_G258V, α_1_L300V and β_2_L296V mutants, we encountered a technical problem. Because of a very small percentage of one of the components (the shut fastest one), the HJCFIT fitting algorithm either failed to converge or tended to yield extreme (and largely random in successive trials) values of the rate constants, especially for the preactivation parameters (δ_2_ and γ_2_). In order to bypass this problem, we performed the optimization in two stages. First, we simplified the distribution by subtracting the slowest shut component using the critical time (*t*_crit_) criterion.^32^ This choice was made based on consideration of the fact that the slowest component for the intracluster events is attributed to the desensitization transitions^23^ and therefore, the data for such a “truncated” shut time distribution were fitted with a simplified model without the desensitized state (Figure 8C). In these conditions, the fit was highly reproducible. Then, such obtained rate constants δ_2_ and γ_2_ were fixed and used in fitting to the full model (Figure 8B) to the complete data (with entire shut times distributions). Fitting of the full data with the complete model with fixed δ_2_ and γ_2_ rates was successful and reproducible. The reliability of this two-step procedure was ultimately validated by confronting the theoretical distributions generated with this modeling with the experimental ones, and as shown in Figure 8E, these distributions overlapped very well. Moreover, when confronting results of such two-step modeling for the WT receptors with data obtained using one-step fitting (Figure 8D), the values of optimized rate constants were statistically indistinguishable (data not shown), further confirming the validity of the two-step approach.

In the case of the α_1_G258V mutant, the flipping rate δ_2_ was unaltered compared to that of the WT receptors, but unflipping γ_2_ was significantly reduced (Figure 8D). In contrast, in the case of the β_2_L296V and α_1_L300V mutants, no significant changes in the preactivation rate constants (δ_2_ and γ_2_) were found. In both α_1_G258V and β_2_L296V mutants the opening (β_2_ and β_2_′) rates were strongly reduced, while the closing rates (α_2_ and α_2_′) were markedly upregulated (Figure 8D). These results on the opening and closing rates appear compatible with particularly evident shortenings of the single-channel openings and prolongation of closures (Figure 7, Tables 2 and 3). Moreover, in these two mutants, a decrease in the desensitization rate *d*_2_ was observed compared to that of WT (Figure 8D) which is in qualitative agreement with weakening of the macroscopic desensitization in these mutants (Figure 3 and Table 1). Taken altogether, the single-channel data modeling revealed two qualitatively different phenotypes: In the case of α_1_G258V, α_1_L300V and β_2_L296V mutations, the predominant effects were weakening of desensitization combined with a strong impairment of opening/closing transitions, whereas for the β_2_G254V mutation, an upregulated desensitization and a moderately affected preactivation were observed.

### Where Is Desensitization Transition Originating from? Bifurcating (Figure 4A) vs Linear Model

The fact that the activation and desensitization gates are located in the close neighborhood on the M2 pore lining segment might suggest that desensitization could originate from the open conformation. This scenario would convert our model from a bifurcating one (Figure 4Aa) to the linear one (R ↔ AR ↔ A_2_R ↔ A_2_F ↔ A_2_O ↔ A_2_D, referred to as the flipped linear model, fLM). To check this possibility, we have tentatively re-examined our modeling for α_1_G258V and β_2_L296V mutants, for which the effects were very clear, both at the macroscopic and single-channel levels using these two simplified schemes. Both models allowed to fairly fit the time course of the current responses mediated by WT and the mutated receptors. However, whereas the bifurcating model (Figure 4Aa) well-reproduced the mean open time for the WT receptors (calculated as 1/α_2_, 0.85 ms), for fLM, it was 0.38 ms (calculated as 1/(α_2_ + *d*_2_)) which is much shorter than that determined in the experiment (Table 3). Moreover, the most disappointing was the estimation of the mean open time using the fLM model for the mutants: 0.54 and 1.73 ms for α_1_G258V and β_2_L296V, respectively, i.e., markedly longer than for the WT receptors (0.38 ms) estimated for this model which is in sharp contrast with that of the experiment (Table 3). At the same time, the bifurcating model (Figure 4Aa) correctly reproduced shortening of the mean open time for these mutants. This analysis confirms the validity of the bifurcating model (Figure 4Aa), thus reinforcing the notion that the desensitization transition occurs from the intermediate (preactivated/flipped) state rather than from the open conformation.

### Role of the Second and Third Transmembrane Domains Is Not Limited to Desensitization

In the present report we addressed the impact of the mutations localized at the second and third transmembrane domains, close to their intracellular ends. This region was indicated by Gielen et al.^27^ as the desensitization gate and the mutations considered here were also explored by these authors. Our most important finding is that, although the α_1_G258V, α_1_L300V, β_2_L296V and β_2_G254V mutations did affect desensitization, their impact was not specific for this transition. In the cases of the α_1_G258V, α_1_L300V and β_2_L296V mutants, there was additionally a very strong effect on opening/closing and, in the case of α_1_G258V and β_2_G254V, a relatively minor effect on the preactivation transition. In addition to these effects on the receptor gating, we observed only a moderate impact on the agonist potency suggesting a weak effect on the agonist binding step. Thus, our results point to a general conclusion that mutations in this region affect late conformational transitions including opening/closing and desensitization with relatively minor effects on preactivation and agonist binding. Thus, in reference to the desensitization process, our results and those of Gielen et al.^27^ are in agreement, but our study extends previous findings by providing further evidence that structural determinants for various conformational transitions are intermingled and tend not to be compartmentalized.

While drawing the comparison to the study of Gielen et al.,^27^ it is also important to indicate crucial methodological differences with respect to the present study. Most of the data presented by this group concerns GABA_A_ α_1_β_2_ receptors (there is also a comparative set of data on α_1_β_2_γ_2_ receptors and glycine receptors), while our report is dedicated exclusively to α_1_β_2_γ_2_ receptors. In our hands, expression of α_1_β_2_ receptors was much smaller than those containing the γ_2_ subunit which would preclude single-channel recordings. Gielen et al.^27^ based their reasoning on observations of the macroscopic desensitization, whereas in our study, the rate constants for microscopic desensitization (*d*_2_, *r*_2_) and for the other transitions (opening/closing and preactivation) were extracted from the macroscopic and single-channel recordings and modeling. As correctly pointed out by these authors, changes induced by the mutations might affect gating efficacy and thereby influence the macroscopic desensitization without actually modifying the the microscopic desensitization rates. In general, a precise determination of how a considered mutation affects a specific gating property is difficult, because any kinetic feature of the recorded current responses (e.g., macroscopic desensitization, rise time, or deactivation) may depend on all of the rate constants in the considered gating model.^33−36^ In reference to the macroscopic desensitization, this issue was investigated in detail by our group in our study by Szczot et al.^26^ (see Figure 10 in this reference) and also by Macdonald and co-workers.^37^ Most of the studies carried out by Gielen et al.^27^ were performed on oocytes (although a set of comparative recordings in HEK 293 cells was also presented) with a slow solution exchange, whereas in our study, rapid solution “jumps” were used; therefore, it is possible that different components of the receptor gating (especially for desensitization) were observed. An important difference was also that Gielen et al.^27^ implemented a wide spectrum of mutation strategies, including chimeras with various portions of different subunit types as well as pairs of point mutations. This elegant approach offered them an advantage to consider potential interactions between specific localizations, e.g., between the M2 and M3 transmembrane segments, and most data presented in their report concerns either double mutants or chimeric structures. In our report, we considered single substitutions which yielded a clear change in kinetic phenotype, and we focused on precise determination of alterations in gating properties by applying macroscopic and single-channel analysis. The use of these two options of recordings offered an advantage to base our inferences on the complementary analyses which, taken together, reinforced our conclusions. It needs to be mentioned, however, that there were some relatively minor discrepancies in assessment of the gating rate constants from the macroscopic and single-channel data. Not surprisingly, the estimation of the desensitization rate constants (*d*_2_, *r*_2_) from macroscopic nonstationary recordings differed from that based on stationary single-channel measurements for all of the tested receptors. As extensively discussed in our previous reports, the desensitization onset can be clearly observed upon rapid agonist applications, offering a unique opportunity to characterize respective rate constants, whereas in the stationary recordings, this dynamic phase of the desensitization process is not visible; therefore, the reliability of estimation of the desensitization rates is limited.^21,38^ Nevertheless, in the single-channel modeling we reproduced well, at the qualitative level, changes in desensitization observed in the macroscopic recordings (for α_1_G258V, α_1_L300V, β_2_L296V =reduction of desensitization and for β_2_G254V= enhancement). For the α_1_G258V and α_1_L300V mutants, the lack of effect on the flipping rate δ_2_ was consistent in the macroscopic and single-channel modeling, but there were differences for the unflipping rate γ_2_ (Figures 4, 5, and 8). In addition, for β_2_G254V, both δ_2_ and γ_2_ were reduced in the single-channel modeling, and this effect was not confirmed in the macroscopic analysis. The reason for these discrepancies is likely to be related, at least to some extent, to different recording conditions but also to the fact that the macroscopic modeling for extensive models is probably more problematic than that based on the single-channel recordings. Nevertheless, it needs to be stressed that our key conclusions indicating major effects of these mutations on the desensitization and opening/closing transitions are particularly consistent in the two approaches.

It is of note that in our study the α_1_G258V mutation was found to reduce the macroscopic desensitization, while Gielen et al.,^27^ who studied this substitution together with other mutations (e.g., β_2_G254A), reported desensitization acceleration and its increased extent. Most likely these different observations reflect the distinct functional impact of a single and double mutations. On the other hand, the β_2_G254V mutation enhanced desensitization in our experiments, similar to observations by Gielen et al.^27^ Thus, taken altogether, our data are in agreement with those of Gielen et al.,^27^ where the M2 and M3 transmembrane segments play an important role in the mechanisms underlying the desensitization process. The importance of the M2 transmembrane segment (but also of M1) was also emphasized by Bianchi and Macdonald^39^ who considered the GABA_A_ receptor chimeras containing portions of γ2 and δ subunits when examining the structural determinants of various slow and fast desensitization components. The major novelty brought by our present study is that the considered residues in the M2 and M3 transmembrane segments, besides being involved in desensitization, have a nearly equally strong impact on the opening/closing transitions. This finding is not surprising as the presumed opening and desensitization restriction gates within the channel pore are not distant from each other^14,40^ (Figure 1C).

### Structural Determinants of Desensitization, Emerging Concept of Diffuse Gating Mechanism

The major point that, in our opinion, deserves a particular emphasis, is that the region of M2 and M3 segments is not unique for determining either opening/closing (“efficacy”) or desensitization. Several studies by our group and by others clearly indicated that the mutations located close to the agonist binding site may have a strong impact on receptor gating, including the specific effect on efficacy: β_2_F200 (loop C),^24^ α_1_F45 (loop G),^22^ α_1_F64A (loop D),^38^ β_2_E155 (loop B).^25,41^ Another intriguing consequence of the mutations at the proximity of the GABA_A_R agonist binding sites is increased spontaneous activity, further underscoring long-range structural interactions underlying receptor openings (e.g., β_2_E155).^25,28,41,42^ These observations clearly indicate that molecular mechanisms underlying receptor openings comprise not only the areas close to the channel opening gate but also several distant regions, which is best documented by involvement of areas close to the agonist binding sites.

The present study adds an interesting observation that structural elements lying “behind” the channel opening gate (from the viewpoint of LBS) are also strongly involved in the receptor opening/closing transitions. The major point that emerges from our present and previous data is that a similar conclusion concerning widespread gating also holds for structural determinants of the desensitization process. There is no doubt that the M2 and M3 transmembrane segments play an important role in determining GABA_A_R desensitization, but several reports also indicate other structural determinants, practically in all major regions of this receptor. As already mentioned in the Introduction, mutation of the α_1_F45 residue (loop G)^22^ affected practically all gating features of the receptor including preactivation, opening/closing, and desensitization providing another example that structural determinants for these gating transitions are shared. Similar conclusions were drawn in our recent study on the role of the loop C (β_2_F200)^24^ in which the impact on all gating characteristics, including desensitization, was reported. Perhaps the most surprising was our recent observation that the mutation of α_1_F14 and β_2_F31 residues, located nearly at the “top” of the ECD, had again a clear impact on all gating transitions including desensitization.^21^ Moreover, it was shown that the involvement of these residues in the receptor gating depends on intersubunit interactions mediated by these phenylalanine residues, further underscoring long-range interactions in the structural mechanisms underlying conformational transitions. All these observations point to the concept of “diffuse gating mechanism” for all conformational transitions, including desensitization. Clearly, some localizations within the GABA_A_ receptor macromolecule might be more critical than the others for various gating features. As comprehensively reviewed by e.g., Cederholm et al.^1^ and Miller and Smart,^2^ mutations of some residues, e.g. at the ECD-TMD interface are “lethal” for receptor gating, whereas others might rather play a “modulatory” role in shaping distinct gating features of this receptor. Nevertheless, the picture emerges that GABA_A_R activation reflects cooperative actions based on several long-range interactions, and therefore, the molecular determinants of receptor gating need to be studied “holistically” by considering GABA_A_R entire structure. It is important to add that Gielen and co-workers,^27^ when proposing the localization of the “desensitization gate” at the M2 and M3 transmembrane segments, were aware of a possibility of widespread rearrangements of the GABA_A_R macromolecule at the ECD-TMD interface and possibly in ECD, whose movement would be triggered by rigid-body motion of the M2 helices.

Based on structural data, clearly indicating two constriction sites at the channel pore^14,40^ interpreted as the activation gate (between the 16′ and 9′ residues) and desensitization gate (around 2′ residue), a concept of two-gate mechanism has been proposed.^43^ However, recent data on α_7_ homomeric nAChR^44^ indicate that both gates might be related to the desensitized state. Our conclusions generally agree with this idea in the sense that final steps of the activation and desensitization conformational transitions are occurring at specific gates at the channel pore, but these processes, especially their preceding steps, are dependent on long-range interactions comprising vast parts of the receptor macromolecule, reflecting the concept of diffuse gating mechanisms.

It is worth mentioning that, in the case of the structurally related pentameric bacterial channel GLIC^45^ as well as possibly GlyR^46^ and nAChR,^47^ a concerted counterclockwise movement comprising both ECD and TMD was demonstrated, nicely illustrating importance of long-range interactions eventually resulting in quaternary twist and tertiary deformation of these macromolecules.

### Mutations Probably Alter Primarily Interactions within TMD

An important question that arises from our research concerns the molecular mechanisms underlining the effects of the considered point mutations on observed changes in the receptor’s kinetic behavior. Although we have not performed either any in depth physicochemical analysis of altered interactions induced by the considered substitutions or molecular dynamics studies, we believe it is worthwhile to (speculatively) reflect on the nature of the possible underlying molecular scenarios. All of the investigated residues are located at the M2 (pore-lining) or M3 (located in the “inner ring” of the TMD helices bundle) α-helices (Figure 1B). Considering that, on the M2 segment, both opening/closing and desensitization constriction sites are located, it seems unsurprising that changes in these kinetic characteristics are so strongly correlated in our mutagenesis studies. Apart from a possible influence of the mutated residues on general stability and conformation of the TMD helices, an interaction with the membrane cannot be excluded. However, the investigated region is located in the hydrophobic area (see the lack of charged residues in Figure 1C) and between tryptophan residues (often anchoring the protein in the membrane at the level of lipid head groups, Figure 1C). Thus, these hypothetical interactions would be limited to nonspecific, low energy couplings with lipid tails or other constituents of the membrane at the level of the membrane’s inner leaflet. Thus, the scenario that seems to be most probable is that observed effects of mutations are caused by changes of van der Waals interactions in the areas between the TMD helices, which ultimately alter the tightness of the helical bundles, thereby influencing their motions linked to gating, that takes the form of the twisting of the subunits and tightening of the pore.

In conclusion, we provide evidence that mutations of M2 and M3 in the TMD strongly affect late gating transitions including opening/closing and desensitization, only weakly changing preactivation and binding. These findings provide further support to the view that the molecular determinants of distinct conformational transitions are shared, and their molecular mechanisms are structurally widespread, pointing to a concept of diffuse gating of GABA_A_R.

## Materials and Methods

### Cell Culture and Expression of Recombinant GABA_A_Rs

All experiments were performed on HEK 293 cells (European Collection of Authenticated Cell Culture, Salisbury, UK) cultured in Dulbecco’s Modified Eagle’s Medium with 10% FBS and 1% Pen/Strep (Thermo Fisher Scientific, Waltham, MA, US) in a humidified atmosphere with 5% CO_2_ at 37 °C. Cells were transferred from flasks and replated on Poly-D-lysine (1 μg/ml) coated coverslips (Carl Roth, Karlsruhe, Germany) and, after 24 h, transiently transfected with FuGENE HD transfection reagent (Promega, Madison, WI, US) at a 3:1 FuGENE HD:DNA ratio with adenoviral pCMV-based plasmids containing rat cDNA of GABA_A_R subunits. Both for wild-type and mutated receptors, the subunits ratio in the transfection solution was: 1:1:3 (0.5:0.5:1.5 μg) with added 0.5 μg of EGFP encoding plasmid for visualization of successfully transfected cells using the fluorescence illuminator (470 nm wavelength, CoolLED, Andover, UK) mounted on a modular inverted microscope (Leica DMi8, Wetzlar, Germany).

### Electrophysiology

Patch-clamp recordings were performed 48 h after transfection. Macroscopic currents evoked by saturating GABA (10 mM) or nonsaturating solutions (for dose–response relationships only) were low-pass-filtered at 10 kHz and recorded at a holding potential of −40 mV using an Axopatch 200B amplifier (Molecular Devices, Sunnyvale, CA, US) and acquired using a Digidata 1550A acquisition card (Molecular Devices, Sunnyvale, CA, US). For signal acquisition, pClamp 10.7 software (Molecular Devices, Sunnyvale, CA, US) was used. Borosilicate glass pipettes (outer diameter = 1.5 mm; inner diameter = 1.0 mm; Hilgenberg, Malsfeld, Germany) were pulled using a P-97 horizontal puller (Sutter Instruments, Novato, CA, US) and filled with intracellular solution (mM): 137 KCl, 1 CaCl_2_, 2 ATP-Mg, 2 MgCl_2_, 10 K-gluconate, 11 EGTA, and 10 HEPES, with the pH adjusted to 7.2 with KOH. The resistance of the pipettes filled with the internal solution were in the range 3–5 MΩ. As the external saline, Standard Ringer’s solution was used (mM): 137 NaCl, 5 KCl, 2 CaCl_2_, 1 MgCl_2_, 10 HEPES, and 20 glucose, with the pH adjusted to 7.2 with NaOH and the osmolarity adjusted to ∼320 mOsm with glucose. Currents were evoked from outside-out membrane patches or in the whole-cell configuration (lifted-cell mode) with the ultrafast perfusion system based on a two-channel θ-glass capillary (Hilgenberg, Malsfeld, Germany) mounted on a piezoelectric-driven translator (Physik Instrumente, Karlsruhe, Germany), as described in detail by Jonas^48^ and by our group.^26,36,49^ Solutions were supplied simultaneously to the two channels with a high-precision SP220IZ syringe pump (World Precision Instruments Inc., Sarasota, FL, US) and the open tip solution exchange time range 150–300 μs, depending on its size of the θ-glass capillary and the speed of flux.

Recordings of the single-channel activity were performed in the cell-attached configuration at a holding potential of 100 mV using an Axopatch 200B amplifier (Molecular Devices, Sunnyvale, CA, US), and the signal was filtered at 10 kHz with a built-in low-pass Bessel filter. Signals were digitized at the 100 kHz sampling rate by a Digidata 1550B acquisition card and Clampex 10.7 software (Molecular Devices, Sunnyvale, CA, US). Thick-wall borosilicate glass pipettes (outer diameter = 1.5 mm; inner diameter = 0.87 mm; Hilgenberg, Malsfeld, Germany) were pulled using a P-1000 horizontal puller (Sutter Instruments, Novato, CA, US), and the pipet resistance was filled with the intrapipette Ringer’s solution range 8–12 MΩ. For the reduction of noise, the pipettes were coated with Sylgard 184 (Dow Corning, Auburn, MI, US) and fire-polished on a microforge. External (and intrapipette) solution was different from the solution used for macroscopic recordings and contained (mM): 102.7 NaCl, 20 Na-gluconate, 2 KCl, 2 CaCl_2_, 1.2 MgCl_2_, 10 HEPES, 20 TEA-Cl, 14 mM D-(+)-glucose, and 15 sucrose (Carl Roth, Karlsruhe, Germany), dissolved in deionized water with the pH adjusted to 7.4 with NaOH. To achieve a further noise reduction, the amount of the external solution in the dish was kept at a minimal level (0.9–1 mL in dish of 35 mm diameter). Only the recordings of patches with seal resistance > 10 GΩ were considered for the analysis.

All of the chemicals were purchased from Merck (Darmstadt, Germany) unless stated otherwise.

### Data Analysis

Macroscopic current traces were analyzed in terms of the amplitude measurements and the current kinetics. The protocol used to construct dose–response relationships for current amplitudes was chosen to avoid any distortion by the current rundown. Each recording started with acquisition of 2–3 sweeps of current responses to saturating [GABA] (10 mM); then a current elicited by a nonsaturating [GABA] was recorded and then, again, a response to saturating [GABA] was acquired. The relative response (nonsaturating/saturating) was calculated. Due to this time-consuming protocol, typically, up to two nonsaturating concentrations could be considered when recording from a single cell. Thus, a complete dose–response relationship was constructed from data collected from different cells. In the dose–response relationships presented in Figure 2, the data for each concentration were obtained from at least 3 cells. The outcome of our dose–response analysis for a specific receptor type is one set of parameters (EC_50_ and *n*_h_) obtained from fitting the Hill equation (eq 1) to the dose–response relationship constructed from mean values of relative responses.
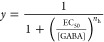
1where y is the value of the relative current amplitude, EC_50_ is the halfmaximal effective concentration, and *n*_h_ is the Hill coefficient. In the present report, comparison to the WT dose–response relationship was made based on the Hill’s curve determined by our group in a recent study,^30^ based on recordings on the WT receptors carried out in the same experimental conditions. The kinetic analysis was performed for current traces recorded in the majority in the excised-patch configuration that assured the best temporal resolution due to fastest solution exchange of the ultrafast perfusion. For the β_2_G254V mutant, excised-patch current recordings were difficult to obtain because of small amplitudes; therefore, recordings for this mutant were performed in the whole-cell configuration (lifted-cell mode) and were compared to a set of analogous control measurements for the WT receptors. A long (500 ms) agonist application protocol was used. The current onset was assessed as the 10–90% rise time (RT). Macroscopic desensitization was described using biexponential fitting, yielding two kinetic time constants (fast and slow, denoted *τ*_fast_ and *τ*_slow_) with respective amplitudes, *A*_fast_ and *A*_slow_, and a constant value *C* (representing the steady-state of current fading, which is a measure of nondesensitizing current) (eq 2)
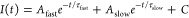
2The percentage of each desensitization component was calculated by means of normalization: %*A*_fast_ + %*A*_slow_ + %*C* = 1. Additionally, to describe the macroscopic desensitization at distinct time points, we used FR10, FR300 and FR500 parameters which describe the fraction of a total amplitude remaining after 10, 300 and 500 ms, respectively. Deactivation kinetics, a phase of current relaxation after agonist removal, was described in terms of a mean time constant (τ_mean_), calculated for either a single exponential fitting or a sum of two exponential components, following eq 3
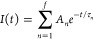
3Analogous to eq 2, *A*_*n*_ is the amplitude of the *n*-th component, and *τ*_*n*_ is the respective time constant. When deactivation was fitted with two exponentials, the mean deactivation time constant was calculated as *τ*_deact_ = *A*_1_%τ_1_ + *A*_2_%τ_2_, where *A*_*n*_% is the percentage of the respective component (*A*_1_% + A_2_% = 1).

Single-channel data analysis was started with the predominant mode selection. Typically, traces exhibited 1–3 modes of activity.^22,23,50^ Event detection analysis in Clampfit 10.7 software (Molecular Devices, Sunnyvale, CA, US) was used to assess open probability (*P*_open_) for distinct clusters, three modes for β_2_G254V = 0.66 ± 0.08, 61% (predominant); 0.90 ± 0.05, 26%; and 0.16 ± 0.12, 13%; and 3 modes for β_2_L296V = 0.20 ± 0.05, 60% (predominant); 0.41 ± 0.04, 19%; and 0.05 ± 0.01, 21%. For α_1_G258V and α_1_L300V, only one mode of activity was present, *P*_open_, for α_1_G258V = 0.33 ± 0.08 and for α_1_L300V = 0.19 ± 0.01. The predominant mode for WT activity was identified according to the description by Lema and Auerbach^50^ and Kisiel et al.^23^ and had the *P*_open_ value of 0.69 ± 0.04. Selected clusters of predominant mode activity were filtered to reach a signal-to-noise ratio of at least 15. The final cutoff frequency (fc) was calculated as follows:
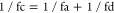
4where fa is the analogue filter frequency (typically 10 kHz) and fd is the digital frequency (offline filtering with 8-pole low-pass Bessel filter by pClamp software). The sampling frequency, fs, was reduced to fs = 10 × fc. Recordings with multilevel openings were excluded from the analysis. The clusters were idealized by a time-course fitting procedure using SCAN software from the DCprogs software pack that was kindly provided to our group by Dr. David Colquhoun. Processed data was used to generate distributions of apparent shut and open times using EKDIST software (DCprogs) for 8000–10 000 events obtained from the idealization. Distributions were fitted with a sum of exponential components (in figures, %*P* = percentage, τ = time constant) which, for the β_2_G254V mutant and WT, was typically four exponentials of shut times and two of open times. The shut time distributions for α_1_G258V, α_1_L300V, and β_2_L296V mutants exhibited three exponential components, two predominant and the third (the fastest one) with a markedly lower percentage.

Bursts analysis was performed by considering the critical time (*t*_crit_) calculated from the shut times distribution analysis using EKDIST, according to the Clapham & Neher criterion^51^ applied to the third and fourth shut time components for β_2_G254V and WT and to the second and third shut time components for α_1_G258V, α_1_L300V and β_2_L296V. This analysis provided information about the open probability for clusters (*P*_open_) and the burst length.

### Macroscopic Data Modeling

Kinetic modeling of macroscopic current responses was based on the frame of the WT flipped Jones-Westbrook Model proposed by Szczot et al.^26^ In the first step, multiple models were created using in-house Python scripts in order to find changes in rate constant essential for depiction of observed changes in currents kinetics of respective mutants relative to WT. Afterward, once the results from the previous step were used as initial guesses, the optimization of the model rates for each current recording (average of all sweeps from single cell) was performed using “waveform fitting” module of ChannelLab software. Because the dose–response relationships for mutants only slightly differed from that for the WT receptors (Figure 2), suggesting little effect on the binding step, the rates *k*_on_ and *k*_off_ were fixed at the WT values^26^ for WT and all of the mutants. In the case of WT, α_1_G258V and β_2_L296V, the fit was done in an ∼30 ms window (see model simulations for details). The same window was applied for β_2_G254V mutant, but because these currents were recorded in the whole-cell configuration, a separate modeling was done for currents mediated by the WT receptors and measured in these experimental conditions. Because in the responses mediated by α_1_L300V only the slow macroscopic desensitization component was affected, fitting was done in an ∼1000 ms window for the model with two desensitized states, and for comparison, the same analysis was carried out for the WT receptors (see model simulations for details).

### Single-Channel Data Modeling

For kinetic modeling of single-channel activity of WT and the β_2_G254V mutant, a framework of kinetic model (two open and two desensitized states) from Kisiel et al.^23^ was adapted. Modeling was performed using HJCFIT software (DCprogs), which is based on the maximum likelihood method that enabled optimization of the rate constants, with the time resolution of 40–90 μs. Kinetic modeling for the α_1_G258V, α_1_L300V and β_2_L296V mutants, due to the reduced number of components in shut time distributions, was based on the kinetic scheme from Kisiel et al.^23^ that was reduced to only one desensitization state. The models for WT and all of the mutants were validated by model simulations at both experimental and 0 μs resolutions and were considered satisfactory when simulated distributions reproduced well the parameters of distributions obtained from the experimental data.

### Structure Visualizations

In each structural visualization, the structure of human GABA_A_R α_1_β_2_γ_2_ subtype in complex with GABA (pdb code 6X3Z)^14^ was used. All of the molecular graphics were prepared in VMD.^52^

### Statistical Analysis

The statistical analysis and data presentation was performed using SigmaPlot 11.0 software (Systat Software). The data were analyzed with Grubb’s test for outlier identification. The single comparisons of a parameter for one mutant vs WT were based on the Student’s *t* test or, alternatively, the Mann–Whitney U test for the data that failed the normality or equal variance test. Values of *p* < 0.05 were considered statistically significant.

Data are presented as mean values and ±SEM in bars and scatter plots.

## ABBREVIATIONS

ECD: extracellular domain
EGFP: enhanced green fluorescence protein
FR10/300/500: fraction of current trace remaining after 10 300 or 500 ms relative to a full current amplitude
LBS: ligand binding site
GABA_A_R: γ-aminobutyric acid type A receptor
[GABA]: concentration of GABA agonist
M1-M3: transmembrane helices of GABA_A_R
pCMV: adenoviral vector with the promoter for cytomegalovirus
pLGIC: pentameric ligand gated ion channel
TMD: transmembrane domain
WT: wild type of the α_1_β_2_γ_2_ GABA_A_
